# Reference Ranges for Serum Uric Acid among Healthy Assamese People

**DOI:** 10.1155/2014/171053

**Published:** 2014-01-09

**Authors:** Madhumita Das, N. C. Borah, M. Ghose, N. Choudhury

**Affiliations:** ^1^Biochemistry Lab, GNRC Hospitals, Dispur, Guwahati 6, 781006, India; ^2^GNRC Lab Services, GNRC Hospitals, Dispur, Guwahati 781006, India

## Abstract

This study was designed to establish reference ranges for serum uric acid among healthy adult Assamese population. Samples from 1470 aged 35–86 years were used to establish age and sex related reference range by the centile method (central 95 percentile) for serum uric acid level. There were 51% (*n* = 754) males and 49% (*n* = 716) females; 75.9% (*n* = 1115) of them were from urban area and the rest 24.1% (*n* = 355) were from the rural area. Majority of the population were nonvegetarian (98.6%, *n* = 1450) and only 1.4% (*n* = 20) were vegetarian. The mean age, weight, height, and uric acid of the studied group were 53.6 ± 11.3 years, 62.6 ± 10.5 kg, 160 ± 9.4 cm, and 5.5 ± 1.4 mg/dL, respectively. There is a statistically significant difference in the mean value of the abovementioned parameters between male and female. The observed reference range of uric acid in the population is 2.6–8.2 mg/dL which is wider than the current reference range used in the laboratory. Except gender (*P* < 0.0001), we did not find any significant relation of uric acid with other selected factors.

## 1. Introduction

Serum uric acid (SUA) is the major end product of purine metabolism in humans; and the level of SUA is rigorously controlled by the balance between uric acid production and excretion [1]. A number of previous studies have reported the relationship between hyperuricemia and various cardiovascular diseases and their risk factors, including metabolic syndrome (MS). According to the earlier studies, not only frank hyperuricemia but also SUA levels almost within the normal range showed a positive correlation with MS [2, 3]. Recent studies have shown that SUA level is significantly associated with nonalcoholic fatty liver disease [4–6].

The current “reference” or “normal range” set for hyperuricemia often fails to identify patients with potential metabolic disorders [7]. The recommended criteria to be used for selection of subjects as source for reference values, the description of the specimen collection conditions [8], and the statistical terminology for description of these values have recently been outlined in human medicine [9]. The approach in developing reference values has been regarded as an important step towards clinical interpretation of laboratory data [10]. The clinician must of course weigh together the history, clinical signs, disease incidence, and so forth with the laboratory data [11–13]. Most of the medical laboratories used to quote “normal ranges” not so related, but ideally test results of the biochemical parameters should be referred to as a population whose status is defined [14, 15].

The reference range of a particular parameter is defined as the concentration of that parameter in a group of clinically healthy persons [16]. Under normal physiological conditions, haemostatic mechanisms keep these parameters within a certain limit [17]. In healthy individuals they vary considerably in different populations [18]. In body fluids and in the absence of diseases, they are influenced by several factors such as age, sex, dietary habits of the people, geographical location, and climate [19–24]. Further, several of these parameters show diurnal and seasonal variations; administration of drugs or vaccines for therapeutic purposes or clinical trials can also cause significant variations [25]. They may also change as a result of variation in techniques used by different laboratories [26–28]. The parameters can also vary following pathological conditions that affect major body organs and systems that produce, secrete, or store them such as the liver, pancreas, kidney, bone marrow, and the immune system [29–31]. In clinical chemistry, reference values are commonly based on reference of the Western population; these usually do not match with the Indian population. As reference values are used by clinicians for interpretation of the results of measurements, it should correctly represent a defined group of population which should have close similarity with the patients under treatment coming for investigation. Since till date no well-documented reference values of uric acid in Assamese population have been established, we planned this study to evaluate the reference values of uric acid of Indian Assamese population, keeping in mind the need for baseline reference laboratory ranges with which to monitor physiological or pathological changes.

## 2. Materials and Methods

### 2.1. Study Population

A total of 2000 subjects of different socioeconomic status from both genders were screened for inclusion in the study. Baseline information on detailed dietary (veg/nonveg), medical and family history along with the information on lifetime use of tobacco (yes/no), alcoholic beverages (yes/no), and current physical activity were collected using a standard questionnaire. Height and weight were also measured with the subjects dressed in a light gown and standing barefoot. The subjects were considered eligible to participate in this study if they met all the criteria after the necessary screening as per the exclusion criteria given in Table 1.

### 2.2. Sampling

12 hrs overnight fasting EDTA, fluoride, and clotted venous blood samples were collected irrespective of seasonal variations throughout the year. Serum and plasma were prepared by centrifugation at 3000 rpm for 15 minutes and analysis was done within 4 hrs of collection. Out of 2000 samples analyzed, 1470 were included in the study after eliminating the results as mentioned in Table 2.

### 2.3. Method of Estimation

Estimation of serum uric acid was done by fully automated indirect uricase UV method. This method is a modification of the uricase method first reported by Bulgar and Johns [32], later modified by Kalchan [33]. Uric acid, which absorbs light at 293 nm is converted by uricase to allantoin, which is nonabsorbing at 293 nm. The change in absorbance at 293 nm due to the disappearance of uric acid is directly proportional to the concentration of uric acid in the sample and is measured using a bichromatic (293, 700 nm) endpoint technique. This method for serum uric acid assay separately determines the initial UV absorbance of the reaction solution before uricase action and the background absorbance after the completion of uricase reaction to derive the net absorbance of uric acid [34]. Uricase UV method is resistant to common endogenous interferences, such as ascorbate and glutathione, and is easy to be calibrated in an autoanalyzer. These advantages make it once recommended as a reference method by International Federation of Clinical Chemistry and Laboratory Medicine (IFCC) [35].

### 2.4. Validation of the Method

In-house validation of the method is done according to CLSI guidelines [36, 37] including calculation of precision, bias, verification of linearity, sensitivity, and assay interference. The precision and bias are calculated (Table 3) on 20 replicates over a period of 20 days on QC materials. The linearity is verified and the method is linear up to 20 mg/dL.

The uric acid method was evaluated for interference from hemolysis, icterus, and lipemia according to CLSI/NCCLS EP7-P. Bias, defined as the difference between the control sample (does not contain interferent) and the test sample (contains the interferent), is shown in Table 4. Bias exceeding 10% is considered “interference.”

The analytical sensitivity is 0.01 mg/dL. It represents the lowest concentration of uric acid that can be distinguished from zero.

### 2.5. Quality Assurance/Quality Control

To ensure the accuracy and precision of the test results, all preanalytical, analytical, and postanalytical precautions were taken into consideration. Instruments, personnel, and procedure validation were carried out through an internal quality control (QC) program with the calculation of standard deviations (SD) and coefficients of variation (CV). It gives an overview of the quality control material used for the evaluation of the assay along with the day-to-day coefficient of variation. The laboratory also participated in external quality program (EQAS) with satisfactory results. As a quality assurance measure, all necessary preanalytical, analytical, and postanalytical precautions were taken to ensure that these data were not biased.

### 2.6. Statistical Analysis

All statistical calculations were performed on the Statistical Package for Social Sciences (SPSS) version 7.5 software (SPSS Inc., Chicago, IL, USA). Mean, median, and standard deviation were calculated for normally distributed continuous data. To further quantify the spread of the data, the 2.5th and the 97.5th percentiles have been presented.

For the demographic and biochemical characteristics, the continuous variables were expressed as mean and standard deviation (SD), whereas the categorical variables were summarized into frequency and percentage. All statistical tests were 2-sided and a *P* < 0.05 was recognized as the statistically significant level.

## 3. Results

In the present study, a cross section of the community was selected and, after careful screening, from 2000 individuals selected, 630 of them were excluded for various abnormal biochemical parameters and a total of 1470 individuals were ultimately retained for inclusion in this study.

**Figure 1:**
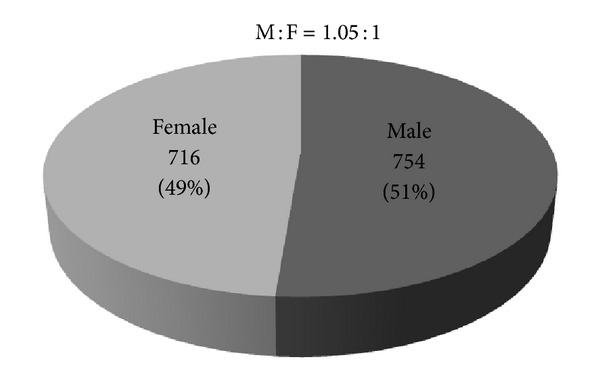
Distribution of gender.

The study sample is 51% (*n* = 754) male and 49% (*n* = 716) female with a male female ratio of 1.05 : 1. Gender distribution of the population is shown in Figure 1.

**Figure 2:**
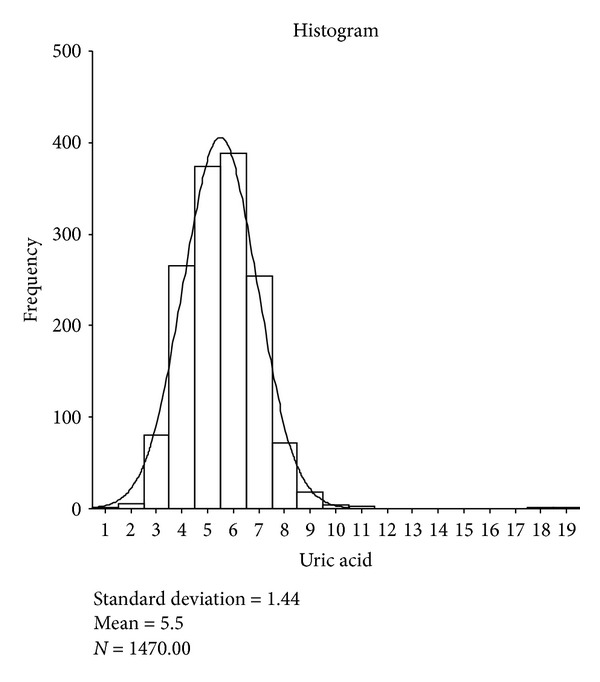
Distribution of uric acid.

The results of the selected characteristics of the target population along with central 95 percentile (mean ± 2 SD) are shown in Table 5 and the distributions of serum uric acid concentrations are shown in Figure 2.

The mean age, weight, height, and serum uric acid level of the entire population are 53.6 ± 11.3 years, 62.6 ± 10.5 kg, 160 ± 9.4 cm, and 5.5 ± 1.4 mg/dL, respectively. The age group of the target population ranges from 35 to 86 yrs. It is observed that all the analyzed parameters show a significantly higher mean value in males than in females. For example, in case of age, male shows a mean of 55.3 years in comparison to female, which is 51.7 years. Similarly, mean values of weight, heights and uric acid in male (64.3 kg, 164.4 cm, and 6.1 mg/dL, resp.) are significantly higher (*P* < 0.0001 in all) than those in female which are 60.7 kg, 155.4 cm, and 4.7 mg/dL respectively. 98.6% (*n* = 1450) of the population are nonvegetarian in comparison to only 1.4% (*n* = 20) vegetarian. Regarding the residential distribution, 75.9% (*n* = 1115) of the study population are from urban area and 24.1% (*n* = 355) are from the rural area.

**Figure 3:**
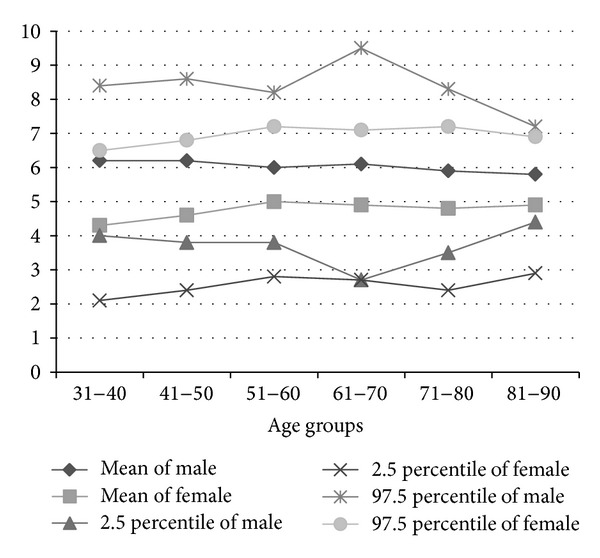
Comparison of results of uric acid between male and female.

The mean, median, SD, and the 2.5 and 97.5 percentiles of uric acid (i.e., lower and upper limits of the central 95 percentile) of the population samples, according to age and gender, are summarized in Figure 3 and Table 6.

It is observed that, as the age advances, mean and median uric acid level also gradually increase except in the age group of 81–90 years, where the number of subjects is very less compared to other classified age groups. The central 95 percentile of the serum uric acid also differs in different age groups and it is found that in the age group of 61–70 years it is much wider, ranging from 2.4 mg/dL to 8.8 mg/dL. In all the age groups, males have higher mean and median values than the females. In the younger age group (31–40 years), males levels exceed those of females by about 2.2 mg/dL. But, as the age advances, this difference starts to decrease. For example, in the age group of 41–50 years, the difference is 1.7 mg/dL, and thereafter it has dropped to almost 1 mg/dL. Results from the female subjects are normally distributed at the age of 31–50 years but skew positively with increasing age. The mean shows an increasing trend showing maximum at the age of 51–60 years and after that it shows a gradual decline. Reverse is observed in men. The mean is more in the younger age group, that is, in between 31–50 years. Performing Chi-square tests (Table 7), we find that uric acid has a highly significant relation with gender (*P* < 0.0001), but it does not show statistically significant relation with age (*P* = 0.684), height (*P* = 0.051), weight (*P* = 0.996), diet (*P* = 0.904), and residential inhabitant (*P* = 0.551).

## 4. Discussion

The recommended procedure by IFCC [38] to identify, collect, and measure enough samples from a sufficiently large reference population is not feasible for most laboratories, which thus have to rely on reference ranges that are based on values obtained in various kits standardized on foreign population, rather than on studies carried out on a well-characterized sample of the population. Keeping in mind all these facts, a sizeable, representative Assamese population both from the urban and rural areas which includes almost all the tribes and ethnic groups that have lived in Assam and met all the necessary criteria is used for the present study.

In the present study, we have found a very wide reference range of uric acid ranging from 2.6 mg/dL to 8.2 mg/dL. In case of male it is 3.5–8.7 mg/dL and in case of female it is 2.5–6.9 mg/dL. Upper limits (i.e., 97.5 percentile) of both of them are greater (by almost 1 mg/dL) than the current reference ranges used in our laboratory (male: 3.5–7.7 mg/dL and female: 2.6–6.0 mg/dL). This may have been caused by the nonstandard selection of subjects. Also the calculated reference range of male of our study is wider than the reference range obtained in a study conducted by Cook et al. in 1970 [39]. It is also observed that males have significantly higher mean value than that of females (*P* < 0.0001) and that is prominent in all the classified age groups. In case of males, there is a little change in the mean value with age, but there is a tendency towards negative skew with increasing age. But reverse is observed in case of females; that is, results from females have a tendency towards positive skew with increasing age. Similar findings are also observed in a study done by Gardner and Scott in 1980 [40]. It is also observed that the mean uric acid in the premenopausal female group is less than that of the postmenopausal group. This rise has been found in some earlier studies also [41–43] and this increase is thought to be attributed to loss of estrogen in the postmenopausal period. Bengtsson and Tibblin in 1974 [44] and McPherson et al. in 1978 [45] compared serum uric acid level between age matched pre- and postmenopausal women and found no significant difference between their uric acid levels. Excluding gender, we did not find any significant relation of uric acid with other selected factors in our study.

## 5. Conclusion

Our study has several limitations such as lack of follow-up of subjects and sample size. We have not studied the effect of racial and socioeconomic status as our purpose was to reflect the condition of the population as a whole. The other shortcomings of the study is noninclusion of age groups below 30 years. The participants of this study were a group of relatively elderly persons, part of which were individuals coming for executive health check-up and another part was from a control group of a study of stroke. A large prospective study including all age groups of various ethnic groups of the North Eastern region will be much more helpful considering that our hospital (GNRC Hospitals, Dispur) is the referral place of the entire North East.

## Figures and Tables

**Table 1:** Exclusion criteria based on history and clinical examination.

Diabetes mellitus	Excessive body weight
Hypertension	Smoking
Cardiovascular disease	Alcohol abuse
Renal disease	Strenuous exercise
Endocrine disorders	Caffeine intake
Liver disease	Any medication
Pregnancy	Use of OCP

**Table 2:** Exclusion criteria based on biochemical test results.

Assessed for eligibility	2000	
Excluded	630	
Eligible for study	1470	

Reasons for exclusion	Number of persons	Reference value used in lab

(1) High blood sugar (>110 mg/dL)	92	60–110 mg/dL
(2) High blood urea (>40 mg/dL)	62	15–40 mg/dL
(3) High serum creatinine (M: >1.3 mg/dL) (F: >1.2 mg/dL)	69	Male: 0.8–1.3 mg/dL Female: 0.6–1.2 mg/dL
(4) Derange LFT (SGOT/AST > 37 U/L) (SGPT/ALT > 65 U/L) (ALP > 136 U/L)	122	SGOT/AST: 15–37 U/L SGPT/ALT: 30–65 U/L ALP: 50–136 U/L
(5) High serum GGT (M: >85 U/L) (F: >55 U/L)	51	Male: 15–85 U/L Female: 5–55 U/L
(6) Lipaemia (high serum triglyceride, >200 mg/dL)	74	30–200 mg/dL
(7) High serum CRP (>0.6 mg/dL)	45	≤0.6 mg/dL
(8) High serum RA (≥20 IU/mL)	40	<20 IU/mL
(9) Low blood haemoglobin (M: <13 gm/dL) (F: <11 gm/dL)	75	Male: 13–18 gm/dL Female: 11–16 gm/dL

**Table 3:** Calculation of precision and bias.

Material used	Assigned mean	Mean obtained in lab	SD	CV	Bias
Bio-Rad QC level 1	4.42	4.35	0.18	4.14	−0.07
Bio-Rad QC level 2	9.03	9.12	0.35	3.84	+0.09

**Table 4:** Assay interference.

Substance tested	Test concentration	Bias%
Hemoglobin	1000 mg/dL	<10
Bilirubin	80 mg/dL	<10
Lipemia	600 mg/dL	<10

**Table 5:** The results of the selected characteristics of the target population.

Criteria	Mean ± SD	Median	Range	Central 95 percentile	*P* value
Age (yrs)					
Total (*n* = 1470)	53.6 ± 11.3	53	35–86	31–76.2	*P* < 0.0001
Male (*n* = 754)	55.3 ± 11.8	56	35–86	31.7–78.9	
Female (*n* = 716)	51.7 ± 10.5	51	35–83	30.7–72.7	
Weight (kg)					
Total (*n* = 1470)	62.6 ± 10.5	62	30–105	41.6–83.6	*P* < 0.0001
Male (*n* = 754)	64.3 ± 10.0	64.7	30–105	44.3–84.3	
Female (*n* = 716)	60.7 ± 10.7	60	30–95	39.3–82.1	
Height (cm)					
Total (*n* = 1470)	160 ± 9.4	160	100–188.5	141.2–178.8	*P* < 0.0001
Male (*n* = 754)	164.4 ± 8.5	165	103–188.5	147.4–181.4	
Female (*n* = 716)	155.4 ± 7.9	155	100–181	139.6–171.2	
Uric acid (mg/dL)					
Total (*n* = 1470)	5.5 ± 1.4	5.5	0.8–19	2.6–8.2	*P* < 0.0001
Male (*n* = 754)	6.1 ± 1.3	6.2	2.5–19	3.5–8.7	
Female (*n* = 716)	4.7 ± 1.1	4.7	0.8–8.9	2.5–6.9	
Diet	*n* (%)				
Nonveg	1450 (98.6%)				
Veg	20 (1.4%)				
Residential areas	*n* (%)				
Urban	1115 (75.9%)				
Rural	355 (24.1%)				

**Table 6:** Statistical analysis of serum uric acid according to age and gender.

Uric acid (mg/dL)	*n* (%)	Mean (SD)	Median	2.5 percentile	97.5 percentile
31–40 yrs					
Male	*n* = 112 (7.6)	6.2 (1.1)	6.4	4	8.4
Female	*n* = 132 (9.0)	4.3 (1.1)	4.2	2.1	6.5
Total	*n* = 244 (16.6)	5.2 (1.4)	5.1	2.4	8
41–50 yrs					
Male	*n* = 153 (10.4)	6.2 (1.2)	6.3	3.8	8.6
Female	*n* = 220 (15.0)	4.6 (1.1)	4.6	2.4	6.8
Total	*n* = 373 (25.4)	5.3 (1.4)	5.3	2.5	8.1
51–60 yrs					
Male	*n* = 219 (14.9)	6.0 (1.1)	6.1	3.8	8.2
Female	*n* = 211 (14.4)	5.0 (1.1)	5	2.8	7.2
Total	*n* = 430 (29.3)	5.5 (1.2)	5.5	3.1	7.9
61–70 yrs					
Male	*n* = 196 (13.3)	6.1 (1.7)	6.1	2.7	9.5
Female	*n* = 128 (8.7)	4.9 (1.1)	4.9	2.7	7.1
Total	*n* = 324 (22.0)	5.6 (1.6)	5.7	2.4	8.8
71–80 yrs					
Male	*n* = 69 (4.7)	5.9 (1.2)	6.2	3.5	8.3
Female	*n* = 22 (1.5)	4.8 (1.2)	4.6	2.4	7.2
Total	*n* = 91 (6.2)	5.7 (1.3)	6.0	3	8.2
81–90 yrs					
Male	*n* = 5 (0.3)	5.8 (0.7)	5.7	4.4	7.2
Female	*n* = 3 (0.2)	4.9 (1.0)	5.2	2.9	6.9
Total	*n* = 8 (0.5)	5.4 (0.9)	5.5	3.6	7.2

Ref. range of uric acid currently used in lab—male: 3.5–7.7 mg/dL and female: 2.6–6.0 mg/dL.

**Table 7:** Results of Chi-square test between uric acid and other parameters.

	Gender	Age	Height	Weight	Diet	Residential area
Uric acid	*P* < 0.0001	*P* = 0.684	*P* = 0.051	*P* = 0.996	*P* = 0.904	*P* = 0.551
